# Pan-Canadian Pharmaceutical Alliance (pCPA): Timelines Analysis and Policy Implications

**DOI:** 10.3389/fphar.2018.01578

**Published:** 2019-02-18

**Authors:** Sam M. Salek, Sarah Lussier Hoskyn, Jeffrey Johns, Nicola Allen, Chander Sehgal

**Affiliations:** ^1^University of Hertfordshire, School of Life and Medical Sciences, Hatfield, United Kingdom; ^2^Institute for Medicines Development, Outcome Research Division, Cardiff, United Kingdom; ^3^Innovative Medicines Canada, Ottawa, ON, Canada; ^4^Global Pricing and Product Strategy, Precision Xtract, London, United Kingdom

**Keywords:** reimbursement, time to list, pan-Canadian Pharmaceutical Alliance, access to new medicines, health technology assessment

## Abstract

This analysis follows our recent study showing that Canadian public reimbursement delays have lengthened from regulatory approval to listing decisions by public drug plans and delayed public access to innovative medicines, mainly due to processes following the Common Drug Review (CDR) and the pan-Canadian Oncology Drug Review (pCODR). Public drug plans participate in a pan-Canadian Pharmaceutical Alliance (pCPA) joint negotiation process before making decisions about whether or not to reimburse a product reviewed through CDR and pCODR. This research aims to report the findings from a comprehensive analysis of pCPA process times, times to reimbursement by public payers in Canada, and to explore the opportunities to reduce total delays in public reimbursement with a specific focus on the pCPA process. An analysis was conducted of pCPA timelines with respect to making decisions about products and indications reviewed through CDR/pCODR, and focusses on three separate time components: time to begin negotiating, time spent negotiating, and time to implement the negotiation (i.e., time to list) in each of nine jurisdictions (i.e., 10 provinces of Canada, excluding Quebec). This study demonstrates the role of post-CDR/pCODR processes in large and lengthening delays to listing new medicines. Notably, oncology products have experienced the longest increases in time to begin negotiating and to complete negotiations. Trends in listing times post-pCPA across provinces are less clear, however, it appears that consistency in terms of timelines across provinces is not happening quite so smoothly for oncology products compared to non-oncology products. Listing rates also appear to be declining for non-oncology products, although this trend is less conclusive for oncology products. Challenges need to be addressed to improve efficiency, transparency, and ultimately reduce pCPA timelines and total timelines to public reimbursement. Suggested ways to improve and streamline the listing process are: (1) transparent target timelines and associated performance incentives for the pCPA and public plan decisions, (2) parallel HTA-pCPA processes to enable pCPA negotiations to start part-way through the HTA review and allow pCPA negotiation information to be fed back into the HTA review, and (3) innovative agreements that consider patient input and earlier coverage with real-world evidence development.

## Introduction

Our recent study, Factors influencing delays in patient access to new medicines in Canada: a retrospective study of reimbursement processes in public drug plans, confirmed that Canadian public reimbursement delays have lengthened in time from regulatory approval (i.e., upon issuance of a Notice of Compliance, NOC) to listing decisions by public drug plans, with increases of 22% in time to list in Quebec, 38% to first provincial listing in any other jurisdiction, and 53% to country-wide listing (Salek et al., 2019). Post-HTA to first provincial listing times, which represents the best-case scenario in terms of the shortest time, increased by 44%. The bulk of the time increase was demonstrated to have occurred during the time period following HTA recommendations by CDR and pCODR - that being the time during which pCPA negotiations and provincial decisions to list are made.

Canada is unique among many Organization for Economic Co-operation and Development countries, being that the nation has universal medicare coverage for hospital and physician services, but not universal drug coverage. As a result, Canada has a collection of provincial and territorial drug plans, and several federal drug plans that cover limited populations in their own jurisdictions. Private drug plans act as a complement to the public drug plans and cover the majority of working Canadians for health services not covered under medicare or public drug plans. An overview of the Canadian public system reimbursement decision pathways for new medicines is provided in Supplementary Figure S1.

In the absence of a single unique payer for pharmaceuticals, the pCPA was established by the Council of Federation (a collective of the provincial and territorial premiers) in 2010. A pan-Canadian body, the pCPA was created for the purpose of conducting joint public drug plan negotiations for innovative and generic drugs in Canada being considered for reimbursement through participating public drug plans. This includes drug plans of all 13 provinces and territories, with the province of Quebec joining in October 2015 and the Federal Government joining in February 2016 (three of the five federal plans participate). The pCPA took several years to be developed, and only became formally established with a permanent, government-funded staff and office in 2015. The pCPA negotiated its first product in 2011. The goal of pCPA negotiations is to achieve “greater value for publicly funded drug programs and patients” (Council of the Federation Secretariat, 2017) by (1) capitalizing on the combined purchasing power of public drug plans across multiple jurisdictions, (2) improving the consistency of medication listing decisions across the country, (3) achieving consistent pricing and lower costs and increasing access to treatment options.

The scope of products under the purview of the pCPA is principally all new drugs having been considered by the national HTA review processes (specifically, the pan-Canadian Oncology Drug Review, or pCODR and the Common Drug Review, or CDR). However, the pCPA does undertake re-negotiations in instances where the terms of a letter of agreement with the pCPA have expired or where there are significant market changes. This negotiation process starts when a collective of public drug program makes a decision, based on the recommendations of CADTH, about whether or not to negotiate for public reimbursement. If the alliance decides to negotiate jointly for the drug, one or two jurisdictions or the pCPA office takes the lead for negotiations with the drug manufacturer. Ontario and Nova Scotia governments tend to take the lead in this process in the context of innovative drugs, while Saskatchewan and Nova Scotia governments lead the process for generics (Council of the Federation, 2014a). If an agreement is reached, a LOI is signed by both the manufacturer and the pCPA, and each participating jurisdiction will subsequently decide whether and when to fund the drug through its own public drug plan under terms agreed to in a confidential product-listing agreement with the manufacturer (Council of the Federation, 2014a; Milliken et al., 2015).

There is a separate review process for innovative medicines and generics. For generic medicines, the pan-Canadian Generic Value Price Initiative, first implemented in April 2014 with generic manufacturers, caps prices relative to the reference innovative product’s transparent list price (Government of Saskatchewan, 2018b). This cap can range from 25 to 85% of the price assigned to the comparator innovator version for the majority of generic medicines, depending on the number of marketed versions. As of April 2018, 48 generic medicines are capped at 18% of the innovator’s price and 20 medicines at 10% of the innovator’s price (Council of the Federation Secretariat, 2017; Government of Saskatchewan, 2018a). Although generic prices negotiated through the pCPA are transparent and generally apply to the entire market, innovator prices negotiated through the pCPA are confidential and non-transparent and only apply to the participating public payers.

Innovative medicines undergo price negotiations before PLAs are implemented and products are listed. From inception to the end of 2017, the pCPA made decisions or undertook negotiations for a total of 309 new innovator products and additional indications: 196 joint negotiations were successfully completed, 20 negotiations were unsuccessful, 36 negotiations were ongoing, and 57 products were not selected for negotiation (pCPA, 2017).

The pCPA is facing many challenges. A backlog of products requiring review began to accumulate in October 2015 and has yet to be resolved. There is also a lack of transparency with respect to review timelines, the negotiation process, and the specific criteria used in decision-making. Furthermore, the provincial implementation (i.e., a PLA) after a LOI is signed is still not guaranteed and timing is unpredictable. For some medicines or indications, pCPA may represent the last step through the reimbursement pathway (i.e., where the product does not make it to listing).

To address the challenges of implementing pCODR drug recommendations, a new committee was formed in May 2016 by the Canadian Association of Provincial Cancer Agencies (CAPCA) called the CDIAC. CDIAC’s role is to supplement pCODR recommendations to the pCPA by “providing advice, ideally prior to the initiation of pCPA negotiations, about how new drugs can be integrated into existing funding algorithms and to achieve greater consistency in drug funding decisions across Canada” (CAPCA, 2018). This process remains extremely non-transparent and there is little publicly-available information about it.

The CDIAC has indicated it will consider trade-offs between maximizing value in terms of patients’ needs and maintaining consistency with pCODR recommendations. While patients and industry recognize the need to promote optimal access and improve implementation of drug funding decisions, they are concerned about added delays in the process. They also emphasized that reforms should not focus only on cost containment (CAPCA, 2017). In their joint submission to CAPCA, Innovative Medicines Canada and BIOTECanada, two associations representing Canadian pharmaceutical manufacturers, raised concerns about the potential for the CDIAC process to overlap with and duplicate some of the elements of the pan-Canadian Cancer Drug Funding Sustainability Initiative (DFSI) (BIOTECanada, 2017). These organizations questioned, for example, to what extent the CDIAC would overlap with some of the work on assessment and implementation currently conducted through pCODR and the pCPA. Although CAPCA intends that CDIAC will operate concurrently with and in a complementary fashion to existing reviews, patients and industry have expressed concerns that it constitutes an additional step in the funding process that will result in further delays in access and to new treatments.

The task of the pCPA is somewhat challenged and complicated by the numerous stakeholders involved, including, among others: payers, including provincial and territorial drug program branches, Federal drug programs, and CAPCA; the regulatory bodies (Health Canada, PMPRB); the HTA bodies (CADTH, INESSS); industry manufacturers (innovative), industry groups (IMC, BIOTECanada, and CGPA); other cross-sector alliances (CACDS, CAPDM, and CPhA), and patient groups (Council of the Federation, 2014b). The pCPA is continuing the development of guidelines and procedures in consultation with stakeholders (pCPAO, 2018).

The guiding principles of the pCPA are somewhat aligned with pCODR principles, being that they strive to be a model of collaboration with industry and patient groups and whereby overall economic impact is considered as “value.” The pCPA reporting structure is different to that of the CADTH; the pCPA reports to the Council of the Federation and is linked to the Health Care Innovation Working Group and the Conference of the Deputy Ministers of Health (pCPA, 2015). The pCPA Executive Group includes the Drug Program Senior Executive Lead from each participating jurisdiction and other executives, directors and managers, who meet twice annually and on an “as-needed” basis to provide strategic guidance through the pCPA Steering Committee, and where applicable, through the Drug Plan Directors and Staff (pCPA, 2015). The pCPA lacks external bodies to ensure its accountability and transparency. In comparison, the pCODR is steered by two advisory bodies: the CADTH PAC, made up of provincial drug program managers, and the PAG, made up of clinical experts within the provincial drug programs. The PAC assists CADTH by giving guidance and strategic advice to ensure that the pCODR process, among other things, “is transparent, timely, fair, effective, and engages key stakeholders” (pCODR, 2016). The PAG provides advice to PAC and pCODR, focusing on operational issues such as input to pCODR to ensure that “recommendations meet the needs of participating provinces/territories and cancer agencies for evidence-based recommendations that guide drug funding decisions” and “may include considerations related to the implementation of recommendations, advice around consultation and information exchange, and information about emerging trends in the development and use of cancer drugs” (CADTH, 2018).

During 2012–2013, IBM Healthcare was contracted under the direction of the Ontario Ministry of Health and Long-Term Care and other partnering provinces and territories to study the process of pCPA price negotiations (Council of the Federation, 2014b). Issues that were given consideration in their report included whether pCPA goals – including non-price goals – were acceptable, and whether a single, pan-Canadian negotiation was better than multiple parallel negotiations. It was noted that provincial subject matter expertise was key to the success of the pCPA, but that resource constraints were limiting the capacity for this in the pCPA context.

Overall, the IBM report (Council of the Federation, 2014b) recommended that the pCPA initiate a “Secretariat Model,” that would see pCPA employees in non-specialized roles working as a team to inform and support the portfolio of drugs being negotiated. Other process-related recommendations included that the pCPA should establish clear time estimates, benchmarks and targets for pCPA processes and subsequently report on the progress of such performance objectives in annual reports. Additionally, it was recommended that the pCPA become more predictable and flexible in its contracting process – including by standardizing LOIs and PLAs – and that it begin to identify complex issues early in the process. It was moreover concluded that pCPA processes could by improved by way of pCPA-led pre-negotiation briefings with manufacturers, HTA bodies and patient groups. Most of these recommendations are yet again the subject of the most recent guideline consultations (pCPAO, 2018).

On September 30, 2014, the pCPA announced that it would establish a secretariat that would be physically located within the Ontario drug plan offices but administered separately (Jeffcott and Manion, 2015). The provinces launched a stakeholder consultation regarding the ongoing development of this new pCPA secretariat, and stakeholder feedback greatly mirrored the recommendations of the IBM report. More specifically, consultation feedback included requests for greater transparency and consistency of process, improvement in timeliness and accountability, improvement in stakeholder engagement (including allowing patient input into the process), as well as other industry-specific asks (pCPA, 2016). It is difficult to determine how many of these recommendations have been implemented.

There is some evidence that the pCPA may be having positive impacts for public payers and patient access to new innovative medicines. For instance, the pCPA has announced that by March 31, 2017, joint negotiations and generic pricing initiatives led to savings to public budgets of $1.28 billion a year, of which $925 million was due to innovative brand agreements, and $355 million was due to generic price reductions (PDCI, 2017a). A previous study found that in the early days of pCPA (2010–2013), there was a decrease in median listing timelines observed in three provinces (Ontario, Manitoba, Prince Edward Island) for products negotiated through pCPA, a net increase in timelines observed in three provinces (New Brunswick, Nova Scotia, Newfoundland), and no change in British Columbia, Alberta, and Saskatchewan. The proportion of drugs listed that had a completed pCPA negotiation went up in all provinces, excluding Prince Edward Island where it remained the same. Interestingly, only Manitoba saw both a significant reduction in timelines and significant increase in the proportion of listings (Milliken et al., 2015).

Despite the indication that some individual processes across the drug review and funding decision pathway have had some success in reducing their own timelines, Canada continues to experience significant delays in listing of innovative medicines. While parallel regulatory and HTA reviews – that is, pre-NOC HTA reviews – are believed to expedite review processes, overall listing timelines continue to increase and research indicates that this is likely attributable to processes post-HTA, particularly for oncology products (Salek et al., 2019). The aim of this research, therefore, was to conduct a comprehensive analysis of pCPA processing times and times to reimbursement by public payers in Canada. This analysis is complemented by an exploration of opportunities to reduce total delays in public reimbursement, with a specific focus on pCPA process.

## Materials and Methods

### Study Design

Almost all public drug plans participate in the pCPA joint negotiation process before making decisions about whether or not to reimburse a product that has been reviewed by CADTH. We conducted an analysis of pCPA timelines for products and indications reviewed by CADTH, focusing on three separate time components: time until negotiations are commenced; time spent negotiating; and time to implement the negotiation (i.e., time to list) in each of the jurisdictions that participate in pCPA review, except for Quebec and the federal drug programs. These were excluded from our analysis given that they joined pCPA later in the course of our study period. The study design for this research followed the STROBE Initiative’s recommendations for reporting observational studies (von Elm et al., 2014). A study period of the beginning of 2014 through to the end of 2016 was chosen given the availability of pCPA data during this timeframe.

### Data Sources

Data was collected by IQVIA to June 2016, and data to December 2016 was supplemented by the authors. Information from the CADTH website was used to identify CADTH recommendations for all new products and indications issued between 2012 to December 2016 (including drug plan submissions), which were then cross-referenced with the Health Canada NOC database (Health Canada, 2017) in order to obtain the respective NOC date. Information was furthermore drawn from the pCPA website to identify negotiation status and outcomes up to the end of December 2016. Provincial plan listing data was sourced through IQVIA’s various provincial listing databases, and directly from individual provincial drug plan websites.

### Data Analyses

The measure used in our analysis is the mean or average, being careful to include volumes whenever applicable. No adjustment is made for changing volumes since much of the processes are standardized and not meant to be impacted by different volumes.

Separate time segments were established as representative of all relevant negotiation and listing decision points and action points as follows: (i) from CADTH final recommendation to beginning of pCPA negotiation; (ii) from start of negotiation to completion of negotiation (including unsuccessful agreement, where appropriate); (iii) from completed negotiation to public listing in the first of the nine jurisdictions as well as in each of the nine jurisdictions. The average time taken for products to be reviewed in each of these three time segments was calculated. We then conducted a time series analysis of 6 months increments for each of the individual review components.

Subsequently, we calculated the total time taken for the pCPA process, including the time taken by pCPA to start and complete a negotiation. Time to start was calculated as the number of calendar days between the date of CADTH recommendation to the publication date of the first Active Negotiations list a drug appeared in (i.e., start date). For this dataset we excluded products that had already received funding in at least one province, thereby bypassing the pCPA process, even if they had not appeared in the pCPA lists. Time to complete a negotiation was calculated as the number of calendar days from the start date to the publication date of the first Completed Negotiations list a drug appeared in. This analysis was based on all products that had a completed negotiation by December 2016 except for those products that were either still being negotiated or waiting to be negotiated by our December 31, 2016 cut off point. A time series analysis was conducted by 6-months increments, providing additional insight into the timelines at play. We excluded products that had no start date (i.e., never appeared in an Active Negotiations list), and products that appeared in the Completed Negotiations list that was published on January 31, 2014 since these pre-date January 31, 2014, but their actual dates are unknown (older negotiations that were completed in the earlier part of pCPA’s existence, 2011–2013). From its inception to December 31, 2016, the pCPA had completed 129 negotiations, 80 of which were completed during our study period (PDCI, 2017b). Our study included 70 of those 80 completed negotiations.

For the time from completed negotiation to listing in the nine jurisdictions, a timelines analysis was conducted for each of the individual nine jurisdictions so as to indicate trends over time in 6-months increments. Changes in listing rates were examined for all products listed by the end of December 2016 that had a completed pCPA negotiation anytime after February 2014 unless otherwise mentioned (with the same aforementioned exclusions, i.e., no start date, and January 31, 2014 Completed Negotiations list).

A drug was considered ‘listed’ if it had a full or restricted listing status, including if it obtained coverage under a special or exceptional access program on the formulary of a provincial drug plan or cancer agency. The absence of a drug name in a given formulary was assumed to mean “not listed.” Time-to-listing for provincial plans was evaluated as the number of calendar days from the date of publication in a completed negotiation to the date the product first appeared in a drug plan’s formulary (usually the date of publication of the newsletter or bulletin published by the drug plan). The exact day, month, and year of CADTH submission and recommendation and of public reimbursement were available from our data sources, whereas only the month and year of pCPA negotiation start and end dates were available (pCPA publishes all lists on the last day of each month). As such, pCPA calculated times could be ±30 days. A sub-analysis of the above timelines was conducted for oncology and non-oncology products, respectively.

## Results

### Total pCPA Time More Than Doubled in a Period of 18 Months While the Number of Negotiations Decreased

**FIGURE 1:**
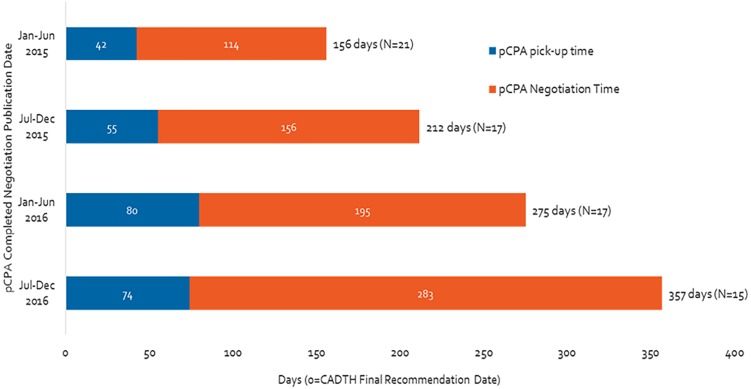
Time from CADTH (CDR and pCODR) recommendation to pCPA completed negotiation publication, from January 2015 to December 2016.

Figure 1 shows the time to start and to complete a negotiation for all products with a completed negotiation in 2015 and 2016. Both the time to start and the time to complete a negotiation increased from 2015 to 2016 (Figure 1). Notably, the total time taken for pCPA steps took more than twice as long in the second half of 2016 as the first half of 2015 (357 days vs. 156 days, or 129% increase). The time spent in negotiations increased over the same time period to a greater extent than did the time taken to begin negotiations (a 150% vs. a 76% increase, respectively). In contrast, the total number of completed negotiations decreased over time from 21 in the first half of 2015 to 15 in the latter part of 2016.

### Total pCPA Time Has Increased More for Oncology Products Than for Non-oncology Products

**FIGURE 2:**
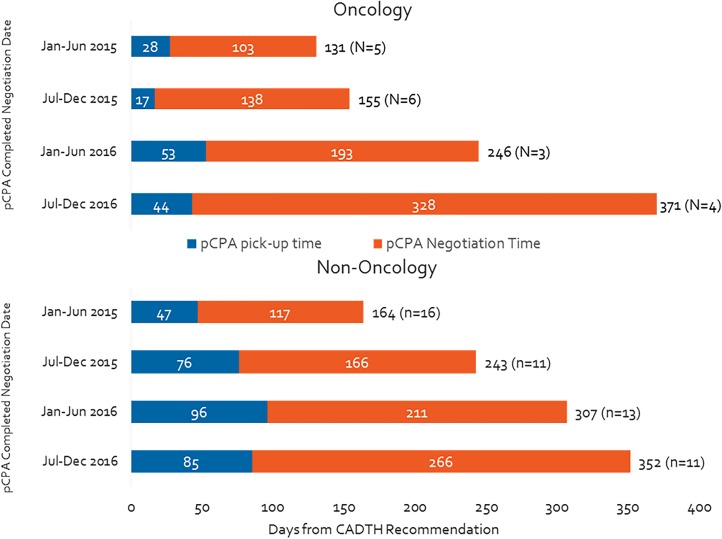
Time from CADTH recommendation to pCPA completed negotiation publication, by oncology and non-oncology products and indications.

Over the duration of our study period, the total time oncology products spent in the pCPA process increased by 180% (from 131 to 371 days), while the time taken for non-oncology products went up by 114% (Figure 2). Moreover, by the second half of 2016, the total pCPA time for oncology products had caught up to and surpassed that of non-oncology products. The number of completed negotiations declined noticeably for non-oncology products (from 16 to 11 from January–June 2015 to July–December 2016), however, remained consistently low for oncology products (from 5 to 4).

### pCPA Is Taking Longer to Start or Decline Negotiations

**FIGURE 3:**
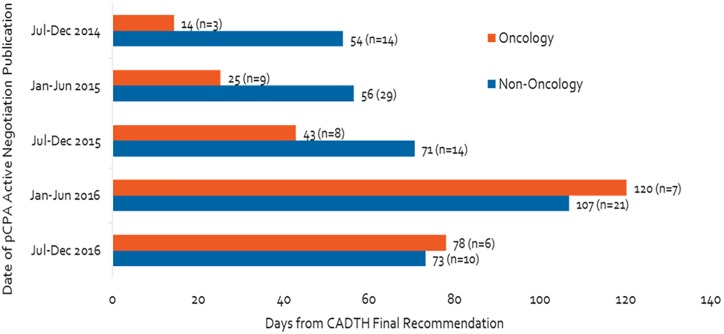
Number of days from CADTH recommendation to start of pCPA negotiation or decision not to negotiate, and number of products.

Figure 3 shows that, from the second half of 2014 to the second half of 2016, the pCPA made a total of 121 decisions on whether or not to negotiate products or indications (33 in oncology and 88 in non-oncology). Time to decision to start or to decline a negotiation has increased 4.5-fold for oncology products or indications, more than non-oncology counterparts, despite steady oncology volumes, from 14 days in the second half of 2014 to 78 days in the second half of 2016, peaking at 120 days in the first half of 2016. Time to decide to start or decline a negotiation for non-oncology products or indications increased by 35% over the full course of our analysis. The time was longer for non-oncology products than oncology products in the second half of 2014, but had become approximately equal for both categories of products in the second half of 2016 (54 vs. 14 days in the second half of 2014, 73 vs. 78 days in the second half of 2016). Volumes have fluctuated significantly for non-oncology products between periods but these do not necessarily follow the same pattern as timelines.

### There Is a Growing Backlog of Products Awaiting Negotiation by pCPA

**FIGURE 4:**
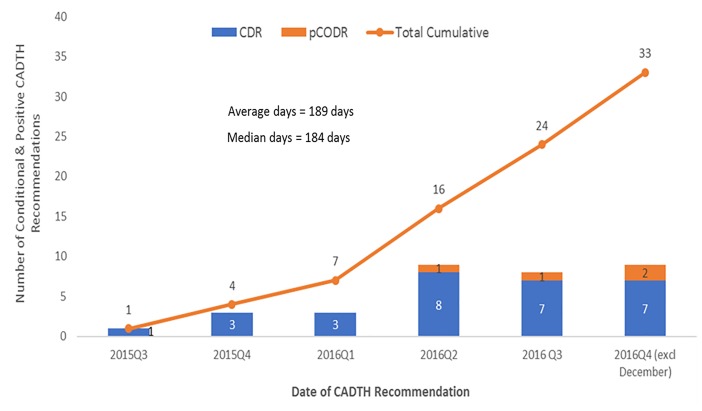
Number of submissions that received a conditional or positive CADTH final recommendation with no negotiation start date or a decision not to negotiate by December 31, 2016.

In total, by December 31, 2016, there were 33 CADTH recommendations (conditional or positive) still waiting for a pCPA decision to negotiate (Figure 4). The average number of days to wait was 189 days and counting (∼6 months), for submissions with no pCPA decision to negotiate by December 31, 2016, and nearly half (16/33) had been waiting longer than 6 months.

### pCPA Negotiations Appear to Be Taking Longer

**FIGURE 5:**
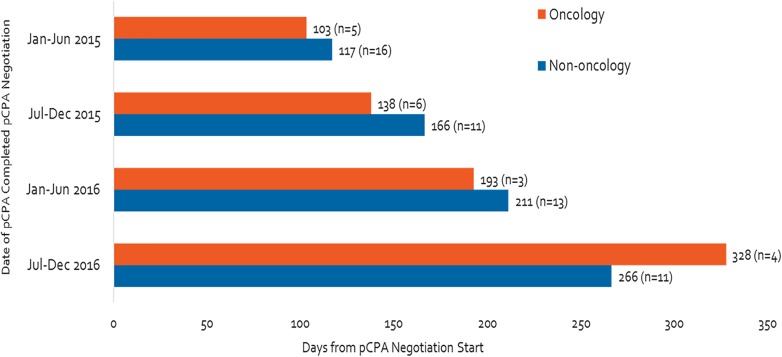
Time of negotiation, oncology products and non-oncology products, between January 2015 and December 2016.

The time spent in negotiation increased considerably between the first half of 2015 and the end of 2016, particularly for oncology products (Figure 5). During this time, oncology product negotiation time increased threefold (from 103 to 328 days), while non-oncology product negotiation time more than doubled (from 117 to 266 days).

**FIGURE 6:**
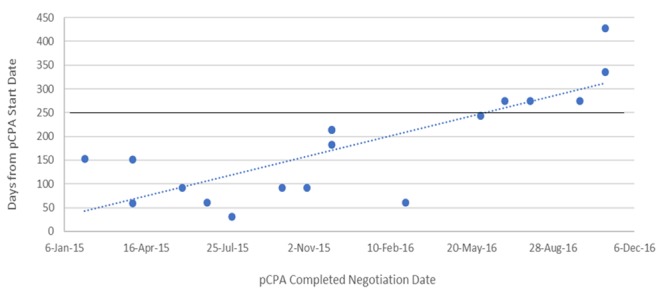
Scatterplot of pCPA negotiation times for oncology products and indications, 2015 and 2016.

**FIGURE 7:**
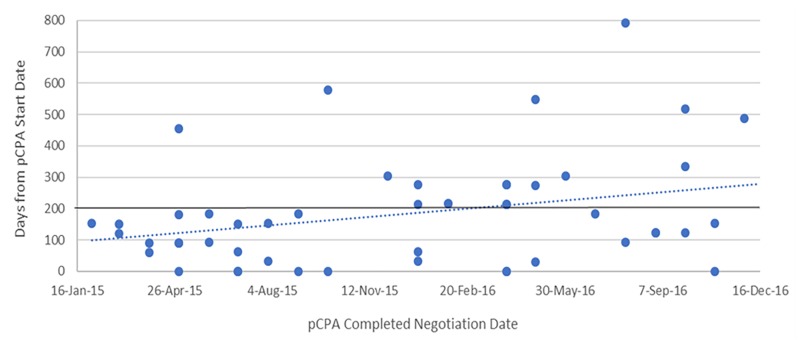
Scatterplot of pCPA negotiation times for non-oncology products and indications, 2015 and 2016.

**FIGURE 8:**
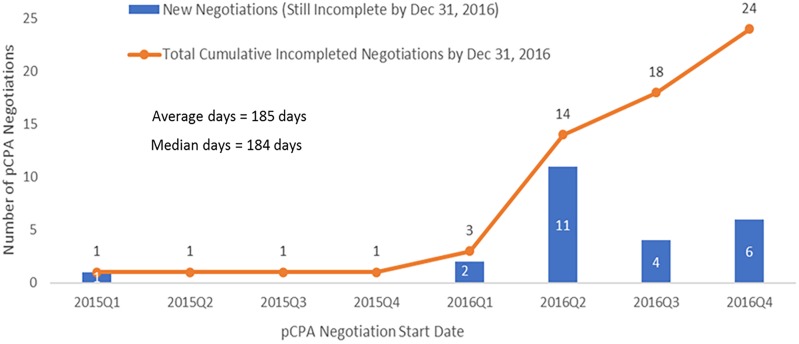
Time spent in negotiation for submissions still incomplete by December 31, 2016 (*N* = 24).

The dramatic increase in times observed in 2016 was not the result of outliers, especially for oncology products (Figure 6). Six of the seven oncology products that had completed negotiations in 2016 took 250 or more days to complete and one of these took over 400 days to complete, while in 2015, nine out of 11 negotiations took 150 days or less to complete and the longest two took 214 days. Whereas nearly all 27 non-oncology products negotiated in 2015 took less than 200 days to be negotiated, more than half of the 24 products negotiated in 2016 took more than 200 days, with four taking longer than 400 days, and one taking nearly 800 days (Figure 7). By December 2016, there were 24 products still under negotiation, and 14 of these had been under negotiation for more than 6 months (Figure 8).

### Provincial Times to List Have Converged Following pCPA Negotiations, but Listing Rates Have Fallen

Over the time period under review, average listing times in individual provinces were long, and were variable across provinces ranging from 495 to 627 days from NOC for non-oncology products (median 582 days), and from 415 to 657 days for oncology products (median 468 days) (Supplementary Figures 2, 3). Time from pCPA negotiation to listing represents a small proportion of total time to listing, however, there is great variability here as well, ranging from 30 to 130 days for non-oncology products (median 69), and from 45 to 360 days for oncology products (median 89 days). Timelines are generally shorter in Ontario, Manitoba, Saskatchewan, Alberta and British Columbia, and longer in New Brunswick, Nova Scotia, Prince Edward Island and Newfoundland and Labrador. Provinces with longer overall times to listing generally are the same provinces with longer post-pCPA timelines. [Note that products vary between provinces based on the unique listings of each province].

**FIGURE 9:**
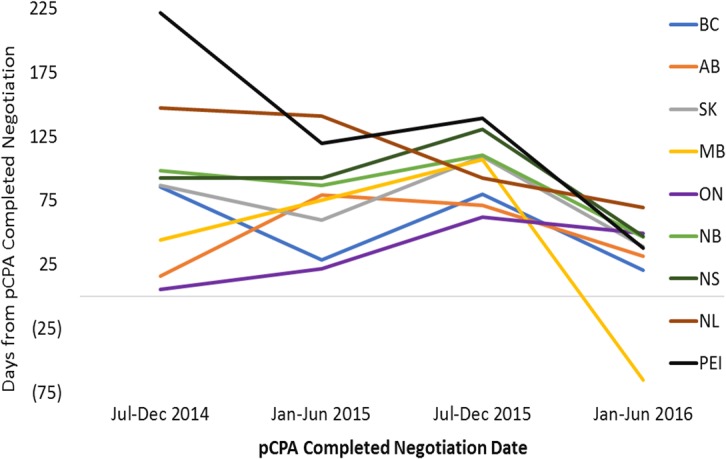
Time to listing from pCPA completed negotiation for non-oncology products and indications that had completed the pCPA negotiation process, by completed negotiation date, for the review period ending on December 31, 2016. AB, Alberta; BC, British Columbia; MB, Manitoba; NB, New Brunswick; NL, Newfoundland and Labrador; NS, Nova Scotia; ON, Ontario; PEI, Prince Edward Island; SK, Saskatchewan.

**FIGURE 10:**
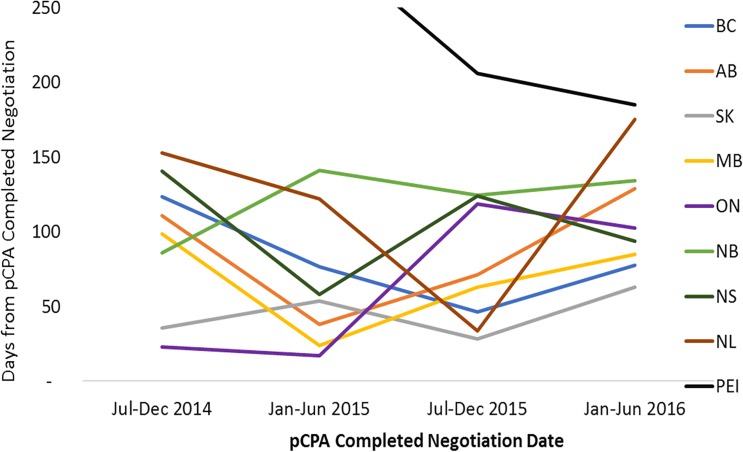
Time to listing from pCPA completed negotiation for oncology products and indications that had completed the pCPA negotiation process, by completed negotiation date, for the review period ending on December 31, 2016. AB, Alberta; BC, British Columbia; MB, Manitoba; NB, New Brunswick; NL, Newfoundland and Labrador; NS, Nova Scotia; ON, Ontario; PEI, Prince Edward Island; SK, Saskatchewan.

For non-oncology products that had completed pCPA negotiation in the first half of 2016, average times to listing had converged to under 100 days in each of the nine jurisdictions (Figure 9). In the majority of provinces, this represented a decrease in timelines post-pCPA over the study period, although the provinces of Ontario and Alberta experienced a net increase in the number of days to listing (44 and 16 days, respectively). For oncology products, no discernible trend can be observed as an outcome of pCPA negotiations in terms of resulting time to list in individual provinces, due to variability across periods (Figure 10).

**FIGURE 11:**
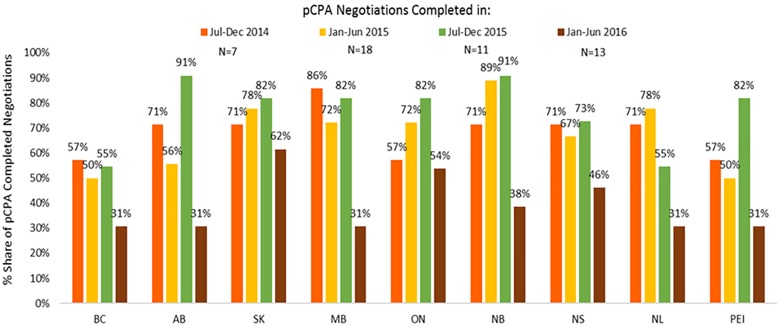
Percentage of non-oncology products and indications negotiated by pCPA that had achieved listing by December 2016, by province. N, number of pCPA LOIs in each respective period. AB, Alberta; BC, British Columbia; MB, Manitoba; NB, New Brunswick; NL, Newfoundland and Labrador; NS, Nova Scotia; ON, Ontario; PEI, Prince Edward Island; SK, Saskatchewan.

**FIGURE 12:**
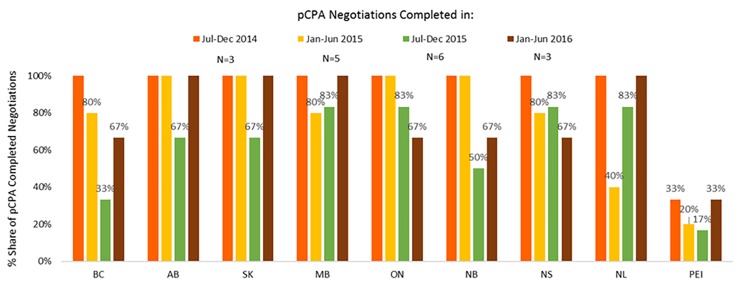
Percentage of oncology products and indications negotiated by pCPA that had achieved listing by December 2016, by province. N, number of pCPA LOIs in each respective period. AB, Alberta; BC, British Columbia; MB, Manitoba; NB, New Brunswick; NL, Newfoundland and Labrador; NS, Nova Scotia; ON, Ontario; PEI, Prince Edward Island; SK, Saskatchewan.

In contrast, listing rates by December 2016 for non-oncology products and indications had declined in all provinces where pCPA negotiations were completed in January–June 2016 as compared to earlier periods (Figure 11). On average, listing rates were 30–40% in the last period under review compared to 60–90% earlier on. For oncology products, listing rates were and remain generally higher than for non-oncology products both initially and at the end of the period under review, and no discernible trend can be observed as an outcome of pCPA negotiations in terms of listing rates in individual provinces due to small numbers (Supplementary Figure S3). However, British Columbia, Ontario, New Brunswick and Nova Scotia all had lower listing rates for products with pCPA negotiations completed in January–June 2016 compared to July–December 2014 (Figure 12).

## Discussion

This study demonstrates the role of the pCPA joint negotiation process in lengthening delays to listing new medicines in Canada’s public drug plans. Notably, oncology products have seen the longest increases in both times to start negotiating and time spent in negotiations.

The cause of the bottleneck observed within the timelines associated with the pCPA is likely to be multifactorial – and may perhaps include a backlog of applications, a lack of human resource capacity, delays in communications between the pCPA and manufacturers, or process or governance issues. Previous individual provincial negotiations may have been faster in some provinces, although would have been subject to duplication and potentially resulting in a different value proposition for different payers. The fact that the pCPA now has to manage and meet the needs of multiple payers with different budget and beneficiary realities may well be resulting in more complex price negotiations and thereby causing delays.

The “buy-in” to pCPA by the provincial drug plans has been good: once a CDR or pCODR review is completed, individual provinces/territories may then negotiate with the manufacturer without pCPA. Alternatively, provinces/territories may negotiate collectively if the pCPA becomes involved and decides that joint pan-Canadian negotiations for the drug will occur. Approximately 91% of positive and conditional CADTH recommendations between January 2012 and December 2015 proceeded to either a pCPA negotiation or an individual provincial negotiation, and approximately 72% of all CADTH recommendations went to a pCPA negotiation (Salek et al., 2019). However, the pCPA seems to be faced with a number of challenges, which ultimately has a substantial impact on delays in patient access to new medicines. It is hoped that the results of this study may identify processes and timelines that require attention and remedial action.

Currently, there are few guidelines in place and a general lack of clarity with respect to the pCPA process – particularly regarding any internal timeline targets governing various steps in the review process. It is not completely clear what criteria are used by the pCPA in determining whether or not to proceed with negotiations for a particular product, although there appear to be signs of prioritization (Salek et al., 2019). It is possible that the increase in pCPA timelines observed in this study and an earlier study (Salek et al., 2019) could be in part due to the lack of target timelines, and the lack of transparent criteria to negotiate may also be creating inequalities in timelines for different medicines.

It is difficult to determine whether the pCPA actually has improved the listing processes and whether it can ever meet all needs of such a diverse public payer community. While it may have reduced the burden on individual provinces, it has increased overall times to listing (Salek et al., 2019), and reduced the number of drugs available (Milliken et al., 2015).

This study also demonstrates that trends in listing times post-pCPA across provinces are less clear, however, it appears that consistency in timelines is not happening quite so smoothly for oncology products compared to non-oncology products. Listing rates also appear to be declining over time for non-oncology products, although this trend was less conclusive for oncology products. This could also be an indication of slower listing times (longer than 6 months) for those products, not captured in our study.

It is possible that the fact that there are different provincial formularies is contributing to the problem of long delays to listing and longer negotiations. Provinces all start the process of making decisions about listing new drug products with different lists of drugs. A recent study by the PMPRB found that out of 729 drugs (molecules) that had been selected for analysis, less than half (48%) were eventually listed in each of the 11 public drug plans analyzed, 34% were listed in at least six (more than half of) plans, and 18% were listed in fewer than six (less than half of) plans (PMPRB, 2018). Despite the fact that terms may be negotiated jointly amongst jurisdictions by way of an LOI, it may be the case that the LOI cannot be implemented uniformly across jurisdictions due to differing budget considerations, willingness to pay and affordability in relation to their respective existing formularies and populations. The United Kingdom offers one example of a national formulary – that is, the British National Formulary (BNF) – that informs formularies in all regions in respect of all products marketed in the United Kingdom. Each region of the United Kingdom has its own Therapeutic Committee that decides what products to reimburse on the basis of their own budgetary considerations, but at a minimum must list those medicines with a positive recommendation from the National Institute for Health and Care Excellence (NICE) within 90 days. Additional medicines listed in the BNF could be selected for reimbursement in individual regions by using an FP10 government prescription form (NICE, 2018). This coordinated, national system seems to have resulted in timeliness and consistency in reimbursement across the country (Curtis and Goldacre, 2018). The mandate letter of Canada’s Federal Health Minister indicates that the creation of a national formulary is being considered, however, no further details are provided about the modalities or timeliness strategies underlying this proposal (Office of the Prime Minister, 2015, 2017).

The situation in Canada is unique, being that there is a centralized regulator, HTA (CADTH, for all provinces except for Quebec) and price negotiation process, but also decentralized formulary decision makers and payers. In comparison, the EU has a centralized regulator in place, but also multiple HTAs, price negotiators, formulary decision-makers and payers.

Although Canada was among the first to introduce the use of economic appraisals and the concept of trade-off and utility in the assessment of pharmaceuticals, the country continues to struggle in terms of its listing and reimbursement processes because the standardized HTA and joint price negotiation processes have not been able to adequately meet the needs of the different payers, and different payers have continued to make reimbursement decisions independently. Following suit, several European countries have recently attempted to work together toward the mutual recognition of HTAs and joint price negotiations to address discrepancies in reimbursement across the region through the BeNeLuxA Initiative. However, BeNeLuxA’s limited experience to date includes a price negotiation that was terminated as the result of the group failing to agree on an acceptable price across the region (BeNeLuxA, 2017). Regardless of this, a made-in-Canada solution can still model an appropriate solution based on the best practices observed in other countries.

The EMA, Health Canada, and CADTH all have mandatory maximum review timelines, which prevent reviews from continuing endlessly. Additionally, CADTH and the EMA both have clear, mandated review milestones setting timelines for both reviewer and manufacturer responses. Moreover, each of these bodies has performance targets with resourcing/funding being tied to performance. EU member states are even mandated to make listing decisions within 3–6 months of EMA marketing authorization (Council of the European Union, 1988), and evidence shows that half of EU states do make decisions within 4–10 months (Millson et al., 2016).

In contrast, public payers in Canada do not have target or mandated timelines for listing products post-marketing authorization, post-HTA recommendation or post-pCPA negotiation. Moreover, neither positive/conditional HTA recommendations nor pCPA LOIs are binding on the public payers. As a result, some recommendations are never followed through with and some LOIs are never implemented or can be delayed – potentially indefinitely. Although our study reveals that time to list following pCPA LOIs has declined for many provinces, listing rates appear to have declined over the same periods which could also be an indication of delayed listing not yet captured in our study. In addition, the poor performance of public reimbursement timelines and listing volumes in Canada compared to the majority of EU states indicate that target timelines for the pCPA and/or for the entire reimbursement system could only benefit the system in terms of timeliness and accountability to patients and manufacturers (Millson et al., 2016).

Given the delays we observed during the pCPA process, we also recommend that pCPA-led pre-negotiation meetings with manufacturers, patient groups and other stakeholders would likely improve the listing process and potentially enable parallel review opportunities.

Salek et al. (2019) revealed that the parallel review opportunity offered by CADTH, called pre-NOC or pre-NOC with conditions (NOC/c) for CDR and pCODR review processes has been largely successful in reducing times from marketing authorization to HTA recommendations and, in some cases, to public reimbursement. The parallel review program has now been expanded to include all classes of drugs – i.e., beyond only drugs with priority review by Health Canada (CADTH-pCODR, 2017), effectively allowing drug marketing authorization and HTA reviews to proceed in parallel for the latter part of the marketing authorization review process, thereby reducing the time to negotiate and eventually make the drug accessible to the public. Since March 2018, all CDR and pCODR submissions can be submitted up to 6 months ahead of the anticipated NOC or NOC/c date, with the potential of completing the HTA review at the same time as the NOC is being given consideration and shaving off up to 6 months in the entire time to list continuum. Although we believe parallel reviews to be advantageous and promising in terms of their ability to reduce time lags and backlogs, there are still barriers to its full adoption and indeed they are under-utilized (pCODR, 2014; CADTH, 2016; CADTH/pCODR, 2018; Salek et al., 2019).

The above noted benefits of a parallel review process raises the question of whether the same concept may be extended to both HTA review and negotiation phases if based on a pre-determined timeline that is similar to that advanced by CADTH. Alternatively, negotiations could commence part-way through the CDR or pCODR process, as applicable – perhaps at a specified time or after some predetermined key analysis milestones indicating that a positive HTA outcome is likely. Currently, negotiations begin after the CDR or pCODR review have been completed, and in fact, there appears to be a growing “gap” in time between when the CADTH recommendation has been published, and when the pCPA negotiation begins. This trend has been especially notable for oncology products, reaching the better part of 1 year by the latter half of 2016. The CDIAC process could be partly to blame, but there is insufficient evidence to draw any conclusions.

The pCPA considers many factors beyond cost effectiveness and product price in its deliberations, including some already given consideration by the anticipated drug plan, such as the treatment landscape, comparators’ prices, and affordability. As pCPA price negotiations unfolded, decisions around pricing could be fed back to the HTA so that it could re-evaluate cost effectiveness, even once therapeutic value had already been shown. The final result would be an HTA recommendation at a cost already negotiated in parallel, which would provide additional certainty to the provinces of the appropriateness of the price point. As an example of this type of scheme, in the United Kingdom, both NICE and the Scottish Medicine Consortium (SMC) enable manufacturers to submit a Patient Access Scheme (PAS) – a confidential discount – part-way through the HTA review, which can increase the possibility of a product obtaining a positive HTA recommendation (ABPI/DOH, 2013). In the United Kingdom, therefore, this provides the added certainty that a positive NICE recommendation effectively guarantees reimbursement across formularies in the United Kingdom. In Canada, such a parallel feedback loop could replace the CDR’s “Do not list at the submitted price” recommendation with a positive “List” recommendation and result in more streamlined pCPA negotiation and public listing processes.

We note that it is promising that Health Canada has begun a consultation on better aligning its own expedited review decision criteria with the rest of the health system. This offers an opportunity for a streamlined pathway from an end to end system perspective, i.e., from NOC to listing.

In addition to the above, there are several new models being explored or implemented in other jurisdictions with the objective of accelerating patients’ access to medicines. In the United Kingdom, the EAMS makes marketing authorizations decisions for products for “life threatening or seriously debilitating conditions” on the scientific opinion of the United Kingdom regulator (MHRA). The EAMS allows for this alternate review process in order to “provide access to Promising Innovative Medicines (PIM) that do not yet have a marketing authorization when there is a clear unmet medical need” (MHRA, 2014; NICE, 2016). In these situations, NICE will commence an HTA evaluation during the EAMS period of 70–90 days before marketing authorization is expected (MHRA, 2014; NICE, 2016). Under this system, the first NICE review Committee decision is published within 3 months of marketing authorization, rather than the usual 6 months. Products recommended by NICE are required to be reimbursed within 90 days of publication of the guidance (MHRA, 2014; NICE, 2016), although this requirement is reduced this to 30 days for EAMS products (MHRA, 2014; NICE, 2016). Consequently, the EAMS process can potentially cut down on more than half of the reimbursement timeline from the time of marketing authorization, while offering a valuable opportunity for early dialog amongst government and arm’s length bodies about product uptake within the NHS (O’Connor et al., 2017). Although limited to specific medicines considered to be in urgent need, the EAMS program demonstrates that faster and more efficient pathways are possible – in particular where parallel reviews are made possible and deadlines are mandated.

In contrast, currently there is no established faster or accelerated pathway for the reimbursement processes that prioritize the medicines that Health Canada has identified fill an unmet need (e.g., priority review products). Except for the pre-NOC HTA process, which could shave off potentially 6 months after NOC in the total time to list, there is no guarantee that the provinces will prioritize those products through the pCPA negotiation process or their own respective provincial listings. In fact, our study demonstrates that total time spent through pCPA negotiation has increased by more than 6 months (201 days) between early 2015 and late 2016, thereby offsetting any potential gains from taking advantage of the pre-NOC HTA process. An earlier study (Salek et al., 2019) demonstrated that products listed in 2015–2016 that had a pre-NOC HTA review still saw a 25% increase in total time to list (84 days) compared to products listed in 2013–2014. The full impact of the worst of the pCPA delays will be felt with 2017 listings.

Italy’s unique early access strategy represented by the Law 648/96 (Ministerial Decree 2003) (Ministero and della Salute, 2017) offers another example of a listing process that provides patients early access to new medicines. This law allows the use of three types of medical products, reimbursed by National Health System: innovative drugs for which the sale is authorized abroad, but not in Italy; drugs which have not yet received an authorization, but have undergone clinical trials; and drugs to be used for a therapeutic indication different from the one which had been authorized (off-label). According to Law 648/96, public health institutions can request early access to medications by submitting a written request to AIFA, underlying the evidence of efficacy as reported in the scientific literature (Balasubramanian et al., 2016; p. 693; Bertozzi et al., 2016). The request is discussed by the Technical and Scientific Commission (CTS) of AIFA and approved for clinical use if deemed appropriate. Thus, the medication becomes available to patients with inclusion and exclusion criteria set by AIFA. In addition, the medication is subject to a program of surveillance and should be reported in a list which is periodically updated.

The study of Russo et al. (2010) investigated the multistep pathway of oncology products during from 2006 to 2008, from the EMA approval, pharmaceutical companies to submit price and reimbursement dossier to the Agenzia Italiana del Farmaco (AIFA), approval by AIFA (the national agency responsible for market authorization of medicines in Italy,) and finally addition to regional/district/hospital formularies and public tender for the purchase of medicines in each Italian region (IR) resulting in patient access. The overall mean time required before patients access was 2.3 years, with the EMA accounted for the greatest proportion of time (31.8%). AIFA approval took an average of 261 days (28.2% of the total time). Highest variability in times, however, was shown by the pharmaceutical companies and Italian regions. Early access was seen for oncology product authorized with a risk-sharing agreement, whereas having a compulsory formulary delayed access.

Navarria et al. (2018) investigated the existing Performance-Based Risk-Sharing Arrangements (PBRSAs) for reimbursements in Italy. PBRSAs are applied to oncology drugs/biologics, and/or high-cost drugs, many with rapid/conditional approval. It was found that savings of only €121 million were made from a total of €3696 million paid. 94.4% of the expenditure was not considered for refund largely because a high percentage of patients had incomplete or interruption of treatment not exempted in the negotiation agreement, as well as health care center inefficiencies “preventing activation of the reimbursement procedure.” A recently introduced “success fee” strategy for performance-based agreements was also described wherein payment is due only for patients who respond to treatment. An ex post payment to the NHS is made for responders, with the cost of therapy for non-responders covered by the drug company. This is a significant change from the previous ex post reimbursement by pharma companies for non-responders with the cost of therapy for responders covered by the NHS at the outset. However, the time to reimbursement was not investigated in this study.

In France, the ANSM, operating under that national public health code, also provides an early access program called Temporary Authorizations for Use (ATU) that allows the exceptional use of medicinal products that have not yet received marketing authorization and that have been subject to clinical trials (Balasubramanian et al., 2016; ANSM, 2017). An important consideration for manufacturers when considering either the Italian or French pathways is that, on one hand – while products will be funded through these mechanisms – the French ATU requires the manufacturer to refund any difference between the ATU price and final negotiated price for reimbursement. On the other hand, the Italian system does not require manufacturers to pay back the difference once the reimbursed price is negotiated.

The Cancer Drugs Fund (CDF) in England affords another example of a program that provides more timely access to the most promising new cancer treatments, and provides better value for money for taxpayers. The CDF was initially set up by the Government in 2011 as a temporary solution to “help patients gain access to cancer drugs not widely available via the NHS,” as they had been rejected by NICE for not being cost effective. After public consultation, in July 2016 the program was amended and relaunched under NICE and NHS England as it was no longer sustainable or desirable (Adcock and Powell, 2015; Hazell, 2015). NICE now issues guidance for coverage with evidence development, essentially becoming a “managed access” fund, paying for new cancer drugs for a limited period of time until they are “definitively approved or rejected by NICE” based on the additional evidence developed. From 2016 onward, each drug in the CDF has evaluation criteria and a timescale for effectiveness to be re-assessed. In contrast, in Canada, there is no managed fund for cancer products. The CDIAC is the most recently formed collective to address the issue of cancer medicines funding, however, so far there is insufficient information of its impact on timeliness and effectiveness in providing access to the most innovative cancer treatments to patients. Given the evidence in our study, oncology products are the most impacted by pCPA delays and overall time to listing.

Currently in Canada, either the CDR or the pCODR may recommend that a drug be listed at a lower price than what was submitted by the manufacturer (if convinced of additional therapeutic benefit vs. comparators but found to not be cost-effective) or to not list at a submitted price (if convinced of similar therapeutic benefit to comparators). Limiting pCPA negotiations to the subject of price alone may contribute to longer negotiations, if the uncertainty is high, and/or if the disparity between the price points of manufacturer and payer is too far apart. Introducing other metrics into the terms of an agreement, such as real-world patient data (RWD) on performance outcomes, physician or patient input, or other health system utilization data could help resolve these complexities, reduce the time spent in negotiations, and improve access to patients from the time of marketing authorization. For example, in Scotland the SMC has the Patient and Clinician Engagement pathway that allows for physician and patient input if the HTA evaluation for an orphan product (drug that treats a rare disease) is heading toward a negative recommendation. In the Canadian context, allowing patient input was indeed highlighted in the IBM report and stakeholder input to consultations in 2014 as a viable means for expediting drug product reviews (Council of the Federation, 2014b; pCPA, 2016).

The problems inherent to providing patients access to innovative medicines at acceptable prices and in a timely way may, in part, be addressed by introducing into listing agreements requirements for developing and applying real-world evidence (RWE). RWE could reduce development costs and become an important means for manufacturers to demonstrate the value of medicines to payers, thus improving the probability of getting reimbursement. Many managed entry agreements or risk sharing deals (an agreement whereby both parties agree to reimburse earlier with conditions to revisit with post-market data) in Europe are based on real world data (RWD). Risk sharing schemes are also common in Italy, for example, to secure reimbursement for cancer treatment, and registries are in place to track outcomes (Pessina et al., 2006). Likewise, the EMA is thinking about fast-tracked, or faster licensing with ongoing submission of RWD during the regulatory review (Bruce, 2016; Cave, 2016). However, at the current scale of data infrastructure and experience with RWE, and of level of resourcing, payers and manufacturers alike mostly resort to price and volume types of discounts.

Another constraint in Canadian review processes is likely attributable to the fact that, after a LOI is signed, the participating jurisdictions either work with the manufacturer on a product- and jurisdiction-specific PLA or work on an implementation of the terms agreed to in the LOI. This implementation is not a second negotiation; however, it is an additional administrative step. It is possible that the multiplicity of interests and actors at play, involving different manufacturers across varying jurisdictions, could be contributing to additional delays in translating the terms of the LOI into contractual form. This results in inconsistent country-wide listings, which the pCPA process is not designed to resolve.

A move to create a single PLA or a standard PLA template for all the negotiating jurisdictions would greatly reduce administrative burdens for all parties such as those identified above, while still allowing individual jurisdictions to opt out of or to alter certain standard terms to suit the unique needs of their population. This was one of the recommendations out of the IBM report and subsequent stakeholder consultations (Council of the Federation, 2014b; pCPA, 2016). The advantages for individual jurisdictions of this type of standardized, but customizable agreement would be the resulting balance between efficiency and transparency across jurisdictions and the ability of individual provinces to contract as per their own needs and laws. A set of standardized terms in PLAs, including certain terms that provinces are allowed to vary, should be drafted and communicated to all relevant stakeholders.

### Limitations

Our study had several limitations as a result of lacking or inadequate data being available. Our analysis excludes data for products and indications that had second letters of intent but no associated CADTH review and recommendation. Our analysis also excludes products negotiated by pCPA before January 31, 2014, due to the fact that the pCPA only began publishing active negotiation and completed negotiation lists in real-time after this date and published retroactive negotiations with a posted date of January 31, 2014. Consequently, these are considered to be artificial dates. Moreover, several products with verifiable negotiation completion dates in 2014 were excluded if they had no corresponding negotiation start dates.

Provincial listing data for Quebec was excluded from this analysis, as Quebec only joined the pCPA process in late 2015 and therefore lacked a complete dataset. Furthermore, provincial listing data from other provinces may be excluded for products and indications that were reviewed by way of a specialized program or that were funded on a case-by-case basis outside of formularies or drug program budgets.

The dates used in our analyses should be considered as being ±30 days in terms of accuracy, as actual pCPA negotiation start and end dates are not reported but rather are reported as being the last day of the respective month.

Data limitations also prevented meaningful analyses that could have been relevant to this issue. For example, pCPA has stopped reporting the results of negotiations for products/indications about which it does not ultimately negotiate (i.e., products/indications that are assigned outcomes such as “Do Not Negotiate” and/or “Provincial Negotiations”). These circumstances hinder meaningful or conclusive performance trends analyses for these drugs (i.e., time to decision to not negotiate). Moreover, pCPA does not identify the source of a request for negotiation – whether it be the manufacturer’s HTA submission or initiation by the drug plan, thereby hindering our understanding of trends in how negotiations are prioritized by pCPA. pCPA also does not identify the type of negotiation: re-negotiation for previously-negotiated product, or negotiation for new product/indication – making it difficult to understand the factors affecting pCPA timelines. The pCPA’s own reporting does not reveal its own methodology for reporting their timelines, making it difficult to replicate and re-evaluate. Lack of data and information on cancer drugs reviewed under the CDIAC also prevents us from truly understanding the processes and timelines at play for oncology drugs during the time they spend between pCODR and pCPA. Therefore, for all the reasons outlined above, our timelines analyses may not reflect statistics reported by pCPA.

## Conclusion

The Canadian public drug reimbursement system faces many challenges in reducing delays in listing new drugs, and much of this is attributable to pCPA process. This paper suggests that the pCPA process is fraught with challenges and requires a great deal of support in order to improve its efficiency, transparency, and ultimately to reduce its own timelines and total timelines to public reimbursement in Canada. There are many possible ways to improve and streamline the listing process, but the relative merits of each needs careful consideration, and the possibility that several solutions can be implemented in parallel must be further explored.

Several examples from other countries suggest that there is a way forward to address each of the challenges and ultimately to reduce timelines. We suggest at minimum three key changes to the pCPA process that would contribute toward reduced timelines, and – if implemented concurrently –could potentially cut total timelines significantly. This would be of great benefit to Canadian patients, drug plans, and the health care system. These include:

1. **Transparent and predictable target timelines:** Transparent and predictable target timelines are necessary for both the pCPA process (from end of CADTH recommendation to pCPA negotiation completion), as well as for public plan decisions from NOC (end to end target timelines). This would provide a clear and predictable overall process, and would include guidelines for prioritization. A proper accountability framework would be needed to assess pCPA’s progress. These timelines should also represent a meaningful improvement over the current state, for example achieving at least a 50% reduction in post-NOC timelines.
2. **Parallel HTA-pCPA process:** The possibility of pCPA discussions starting part-way through the HTA review should be explored, in such a manner as to allow pCPA negotiation information to be fed back into the HTA review and recommendation and to further refine the pCPA negotiation agreement.
3. **Innovative agreements that consider patient input and earlier coverage with real-world evidence development:** A process should be developed that would allow patients to input directly into pCPA negotiations as well as to reach earlier, interim agreements that are conditional on real-world evidence development. This could become part of a parallel-review process with HTA whereby real-world evidence would be developed and then both the HTA and pCPA processes could be re-opened and occur simultaneously, without causing Canadian patients to wait for these deliberations indefinitely.

Other changes, such as establishing standardized PLA templates, could also improve the process and reduce timelines.

Despite certain best practices noted from other jurisdiction, more discussion is needed on “made in Canada” solutions, but this at a minimum requires a collaborative approach between review agencies and with industry, payers, and clinicians to help improve the process going forward.

## Author Contributions

SS contributed to study design, data analysis, interpretation of the results, and first draft of the manuscript. SLH contributed to study design, data collection, analysis, interpretation of the results, and manuscript review. JJ contributed to interpretation of the results and first draft of the manuscript. NA review of several drafts of the manuscript. CS contributed to interpretation of the results and review of the manuscript.

## Conflict of Interest Statement

SLH and CS were employed by company Innovative Medicines Canada, Canada. NA was employed by company Precision Xtract, United Kingdom. The remaining authors declare that the research was conducted in the absence of any commercial or financial relationships that could be construed as a potential conflict of interest.

## Supplementary Material

FIGURE S1 A simplified overview of the public system reimbursement decision pathway for new medicines.

FIGURE S2 Days from NOC and from pCPA to listing and number of listings for CDR-reviewed products with pCPA completed negotiation, 2012–December 2016, by individual province. (Products negotiated before 2014 are excluded due to missing data on actual negotiation start and completion dates).

FIGURE S3 Days from NOC and from pCPA to listing and number of listings for pCODR-reviewed products with pCPA completed negotiation, 2012–December 2016, by individual province. (Products negotiated before 2014 are excluded due to missing data on actual negotiation start and completion dates).

## Abbreviations

ANSM: Agence Nationale de Sécurité du Medicament et des Produits de Santé
CADTH: Canadian Agency for Drugs and Technology in Health
CDIAC: Cancer Drug Implementation Advisory Committee
CDR: common drug review
CEDAC: Canadian Expert Drug Advisory Committee
EAMS: early access to medicines scheme
EMA: European Medicines Agency
HTA: Health Technology Assessment
INESSS: Institut national d’excellence en santé et en services sociaux (National Institute for Excellence in Health and Social Services)
LOI: letter of intent
MHRA: Medicines and Health Products Regulatory Authority
NHS: National Health Service
NICE: National Institute for Clinical Excellence
NOC: notice of compliance
NOCc: notice of compliance with conditions
PAC: pCODR Advisory Committee
PAG: pCODR Provincial Advisory Group
pCODR: pan-Canadian Oncology Drug Review
pCPA: pan-Canadian Pharmaceutical Alliance
PLA: Product Listing Agreement
PMPRB: Patented Medicine Prices Review Board
RWE: real world evidence.
