# Supplementary figures and images for: Pan-Canadian Pharmaceutical Alliance (pCPA): Timelines Analysis and Policy Implications
Source: Front Pharmacol. 2019 Feb 18;9:1578. doi: 10.3389/fphar.2018.01578 (PMC6387957; doi:10.3389/fphar.2018.01578)

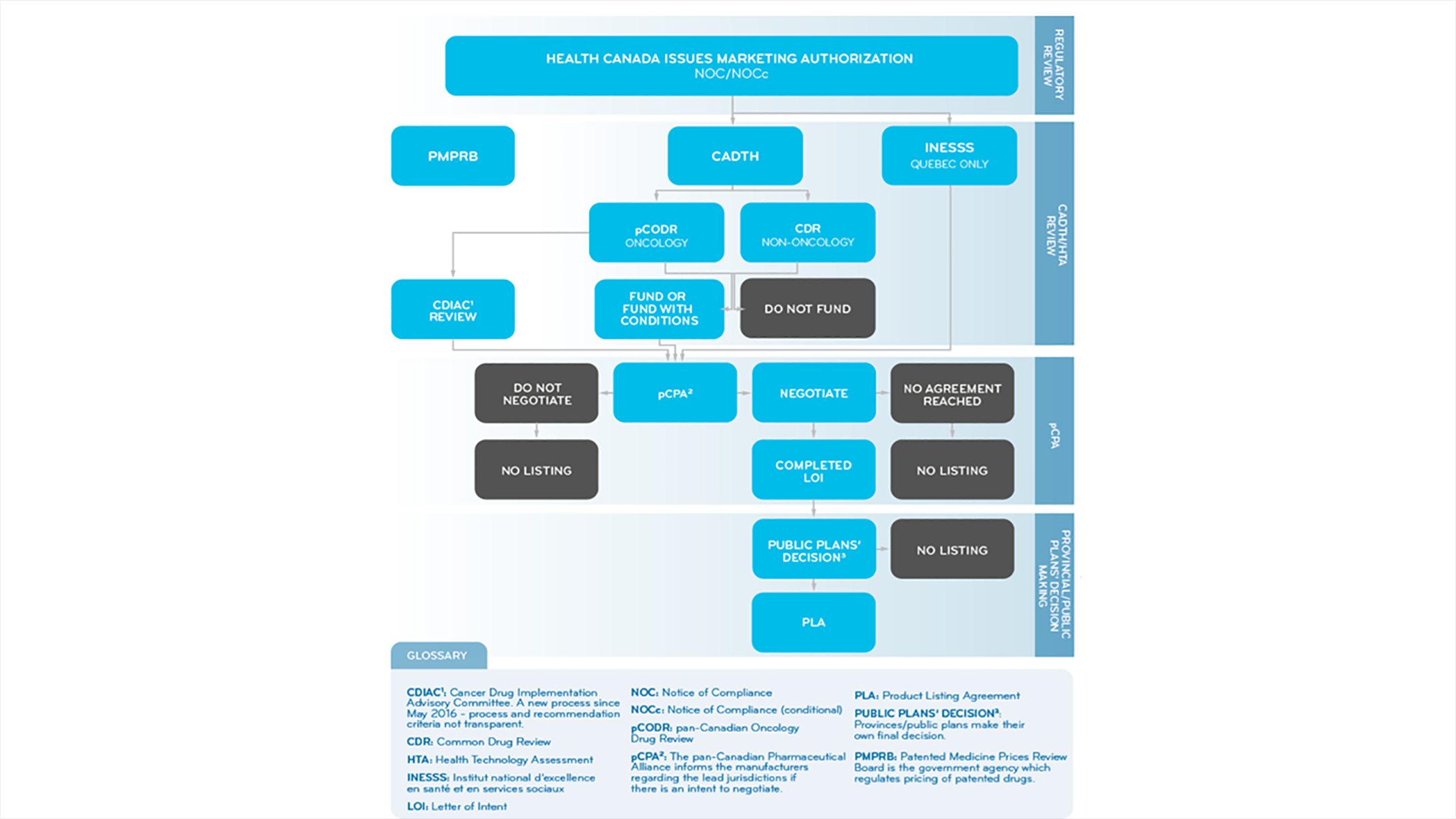

Supplement: FIGURE S1 — A simplified overview of the public system reimbursement decision pathway for new medicines. [file Image_1.TIF]

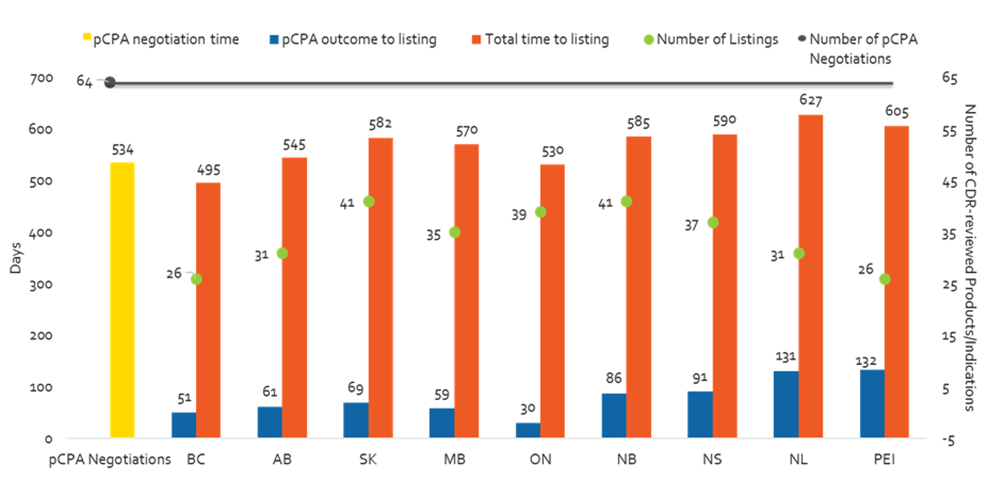

Supplement: FIGURE S2 — Days from NOC and from pCPA to listing and number of listings for CDR-reviewed products with pCPA completed negotiation, 2012–December 2016, by individual province. (Products negotiated before 2014 are excluded due to missing data on actual negotiation start and completion dates). [file Image_2.TIF]

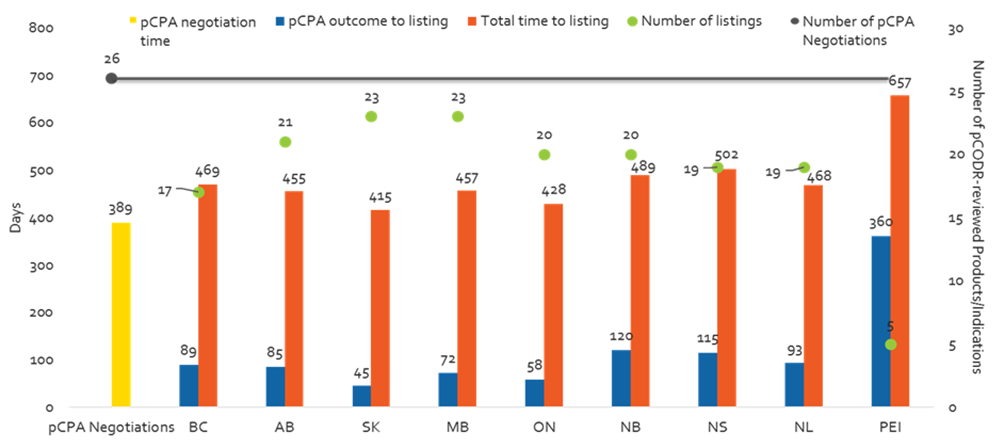

Supplement: FIGURE S3 — Days from NOC and from pCPA to listing and number of listings for pCODR-reviewed products with pCPA completed negotiation, 2012–December 2016, by individual province. (Products negotiated before 2014 are excluded due to missing data on actual negotiation start and completion dates). [file Image_3.TIF]
